# BRCA germline mutations in multiethnic gynecologic patients: A 10-year retrospective analysis from a single cancer institute

**DOI:** 10.1371/journal.pone.0286998

**Published:** 2023-06-13

**Authors:** Christina H. Wei, Susan Shehayeb, Nicole Lugo Santiago, Laura Kruper, Ernest Han, Edward Wang, Mihaela Cristea, Lorna Rodriguez-Rodriguez, Susan E. Yost, Daphne Stewart

**Affiliations:** 1 Department of Pathology, City of Hope Comprehensive Cancer Institute, Duarte, California, United States of America; 2 Department of Population Sciences, City of Hope Comprehensive Cancer Institute, Duarte, California, United States of America; 3 Department of Surgery, City of Hope Comprehensive Cancer Institute, Duarte, California, United States of America; 4 Department or Medical Oncology & Therapeutics Research, City of Hope Comprehensive Cancer Institute, Duarte, CA, United States of America; CNR, ITALY

## Abstract

Histologic and genetic mutation information from racially and ethnically diverse populations is warranted to better inform future cancer predisposition and promote health equity. A single institutional, retrospective capture of patients with gynecologic conditions and genetic susceptibilities to malignant neoplasms of the breast or ovaries was performed. This was achieved with manual curation of the electronic medical record (EMR) from 2010–2020 with the use of ICD-10 code searches. Among 8983 consecutive women identified with gynecologic conditions, 184 were diagnosed with pathogenic/likely pathogenic (P/LP) germline *BRCA* (gBRCA) mutations. Median age was 54 (22–90). Mutations included insertion/deletion (majority frameshift, 57.4%), substitution (32.4%), large structural rearrangement (5.4%), and alteration in splice site/intronic sequence (4.7%). A total of 48% were non-Hispanic White, 32% Hispanic or Latino, 13% Asian, 2% Black, and 5% Other. The most common pathology was high grade serous carcinoma (HGSC, 63%), followed by unclassified/high grade carcinoma (13%). Additional multigene panels led to the detection of 23 additional BRCA-positive patients with germline co-mutations and/or variants of uncertain significance in genes functionally involved in DNA repair mechanisms. Hispanic or Latino and Asian individuals comprised 45% of patients with concomitant gynecologic condition and g*BRCA* positivity in our cohort, confirming that germline mutations are represented across racial and ethnic groups. Insertion/deletion mutations, the majority of which led to a frameshift change, occurred in approximately half of our patient cohort, which may have prognostic implication for therapy resistance. Prospective studies are needed to unravel the significance of germline co-mutations in gynecologic patients.

## Introduction

Approximately 15% of individuals with ovarian cancer and 40% with family history of ovarian cancer have pathogenic *BRCA1* or *BRCA2* germline mutations (*gBRCA1/2*) [1–3]. Current American Society of Clinical Oncology (ASCO) and National Comprehensive Cancer Network (NCCN) guidelines recommend testing all patients with epithelial ovarian cancer, fallopian tube, or primary peritoneal cancers for germline and somatic mutations in *BRCA* genes [1,4,5]. The frequency of mutations may vary depending on patient demographic factors, such as age and ancestry. In addition, there is evidence that testing disparities exist in women with ovarian cancer by different insurance types (private versus government insured), age groups (< 45 years), race or ancestry groups [6,7]. The current published literature regarding genetic variation with BRCA mutations includes a SEER database cohort study that describes the prevalence of *BRCA1* pathogenic variant in non-Hispanic Whites, Blacks, Asians, and Hispanics/Latinos were 7.2%, 13.4%, 6.2%, and 16.1%, respectively [8]. For *BRCA2*, prevalence of pathogenic variant in non-Hispanic Whites, Blacks, Asians, and Hispanics/Latinos were 5.4%, 5.3%, 8.4%, and 5.6%, respectively.

The observed differences amongst different races and ethnicities may be influenced, in part, by testing gaps, since approximately 34% of non-Hispanic white women were tested for germline mutations in the study, in contrast to only 22% and 25% in Black and Latino women, respectively. Risk reducing interventions are a critical component to decreasing cancer morbidity and mortality. If underdiagnosed, certain groups may be at high risk of not taking advantage of these interventions, leading to worst outcomes related to health inequities. Given the significant BRCA germline mutation rate across different races, more research is needed to better inform how certain groups may be at higher risk for certain hereditary cancers, particularly, groups that have been historically underrepresented.

In this article, we performed a retrospective chart review to study a cohort of unselected women with *BRCA1/2* germline mutations who were treated at our institution, which serves a large multiethnic population, for gynecologic conditions from 2010–2020. The objectives of this study are to report these patient-level findings to clarify trends and patterns that may not be captured in large population analyses.

## Materials and methods

This study was approved under City of Hope Institutional Review Board (IRB 21257) for a Waiver of Informed Consent (45CFR46.116(d)) and Waiver of HIPAA Authorization (45CFR164.512(i)(2)(ii)). The authors had access to information that could potentially identify individual participants during or after data collection, but due to the exempt status the identity of patients may not be disclosed. The study encompasses a single institutional, retrospective review of patients with benign and malignant gynecologic (GYN) conditions of the ovary, fallopian tubes, and uterus, as well as g*BRCA1/2* mutation from January 2010 to December 2020. The patients were identified using the electronic health medical records (EHMR) using ICD-10 codes (and related diagnostic codes) that encompassed benign and malignant gynecologic conditions (C56-C57 for malignant tubo-ovarian conditions, Z80.41 for family history of malignant neoplasm of ovary, Z85.43 for personal history of malignant neoplasm of ovary, C79.62 for secondary malignant neoplasm of ovary, D27-D28 for benign tubo-ovarian conditions, and C53-C55 for uterine conditions). These patients were then further refined to those with only *BRCA1* or *BRCA2* germline mutations using different ICD-10 codes (Z31.430 and Z31.440 for encounter for testing for encounter for testing for genetic disease carrier status for procreative management; Z15.01, genetic susceptibility to malignant neoplasm of breast; Z15.02, genetic susceptibility to malignant neoplasm of ovary; and Z84.81 family history of carrier of genetic disease), followed by manual curation for more detailed patient-level data. The data collection was performed by research bioinformaticians. Manual curation was performed by a data honest-broker who consulted with the principal investigators (CHW and DS) for data interpretation-related inquiries. A board-certified genetic counselor (SS) reviewed the genotype for accuracy.

## Results

A total of 8983 patients with benign and malignant gynecologic conditions were identified by retrospective chart review (3362 tubo-ovarian; 6139 uterine; 518 patients with concurrent uterine and tubo-ovarian conditions). Genetic testing selection (e.g., history of ovarian cancer) was performed according to clinical guidelines. From these 8983 patients, we identified 184 cases with g*BRCA* mutations (110 *BRCA1* and 74 *BRCA2*). Our cohort’s median age was 54 (range 22–90). Racial distribution was 48% White, 32% Hispanic or Latino, 13% Asian, 2% Black, and 5% others. Germline *BRCA* mutation prevalence in those with tubo-ovarian neoplasm (benign and malignant) without primary uterine malignancy was 6% (157/2844; rate of testing indeterminant). Most of the *gBRCA* mutations in this cohort were diagnosed by various commercial molecular sequencing tests after 2013 (73%; 125 of 171 patients with known test result date). These included Myriad Genetics (n = 60), Ambry (n = 21), Invitae Genetic Testing (n = 21), Integrated Genetics (n = 7), Quest (n = 6), Color’s Hereditary Cancer Test (n = 3), and others (n = 66).”

### Histologic variation

Amongst patients with g*BRCA* mutations, 170 patient had neoplastic gynecologic histology. The 14 other cases were gBRCA carriers who did not have gynecologic neoplasm (these women were probably tested due to compelling family history of cancer) and were being followed clinically for cancer surveillance. The most common pathology was high grade serous carcinoma (HGSC) of tubo-ovarian origin (70%), followed by unclassified/high grade carcinoma (12%). Other ovarian pathologies were observed, including germ cell tumor (3%), ovarian carcinoma with endometrioid histology (4%), clear cell (0.6%), and transitional cell (0.6%). All germ cell tumors found in this series were teratomas; 4 cases were benign, one case was malignant with somatic squamous cell carcinoma transformation accounting for the malignant tumor component. Serous tubal intraepithelial carcinoma (STIC) without invasive high grade serous carcinoma was seen in 3 patients with *BRCA* alterations (1%).

The most frequent benign ovarian tumor seen in our cohort was mature cystic teratoma (2%; 4 pts), followed by benign serous cystadenoma (2%; 3 pts). Four of the 5 patients with teratomas had germline *BRCA2* deleterious mutations. The median age of teratoma diagnosis was 42 (range 36–43 years).

The racial and ethnic distribution of those with *BRCA* germline mutations and ovarian teratoma includes 2 non-Hispanic White, 2 Asian, and 1 Hispanic or Latino. Uterine carcinoma was identified in 4% of our cohort. There were 3 cases with high grade serous histology (2 pure serous and 1 with mixed serous and endometrioid histology). Of these, two occurred within the setting of a *BRCA1* germline mutation and one in the setting of a *BRCA2* germline mutation. Fourteen *BRCA* positive patients underwent risk surveillance or had risk-reducing bilateral salpingo-oophorectomy with no significant histologic findings. The histologic distribution in patients with *BRCA* germline mutation is summarized in Table 1.

**Table 1:** Summary of gynecologic neoplastic histology observed in women with *gBRCA* mutations (n = 170).

Anatomic Location	Histology	Number of BRCA patients (%)
**Ovary/Fallopian Tube**	High grade serous carcinoma	120 (65%)
	Teratoma (malignant and benign)	5 (3%)
	Mixed serous and endometrioid	4 (2%)
	Serous cystadenoma	3 (2%)
	Carcinosarcoma	1 (0.5%)
	Clear cell carcinoma	1 (0.5%)
	Serous tubal intraepithelial carcinoma	2 (1%)
	Others/unclassified	21 (11%)
**Endometrium**	High grade serous (pure)	2 (1%)
	Mixed high grade serous and endometroid	1 (0.5%)
	Endometrioid	3 (2%)
	Carcinosarcoma	1 (0.5%)
Others (Parametrium, cervix)	HPV-associated neoplasm	6 (3%)

### Mutational variation

**Fig 1:**
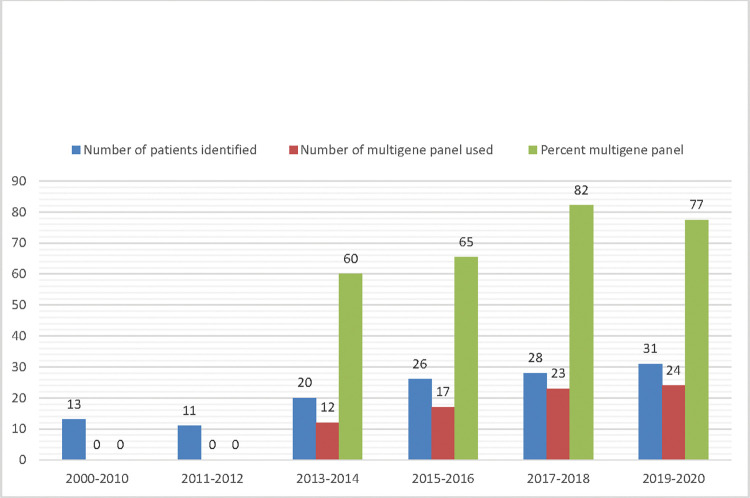
Evolution of use of multigene panel usage and patients identified from 2000 to 2020. An increase in testing and multigene panel usage was observed over time.

Mutational spectrum included insertions/deletions (57.4%), substitution (32.4%), large structural rearrangement (5.4%), and alteration in splice site/intronic sequence (4.7%). Details of variants by type and race-ethnicity are presented in S1 Table. Over time, we observed an increase in testing and multigene panel usage (Fig 1). We identified 10 patients (5 non-Hispanic White, 3 Asian, 2 Hispanic or Latino) with co-mutations in genes functionally involved in homologous recombination repair (*PALB2*, *CHEK2*, one patient each), DNA mismatch repair (*PMS2* and *MLH1*, one patient each), oxidative DNA damage repair (*MUTYH*, 3 patients), Wnt signaling pathway (*APC*, 1 patient), cell cycle (*CDKN1C*, 1 patient), and mitochondrial metabolic process (*SDHA*, 1 patient). Amongst the 10 patients with germline co-mutations, 6 patients have neoplastic histologic information. Of these, three had endometrioid histology (50%) and three had high grade serous histology (50%). We also identified 23 patients (11 non-Hispanic White, 7 Asian, 5 Hispanic or Latino) with VUS in genes functionally involved in homologous recombination repair (*BRCA1*, *BRCA2*, *CHEK2*, *BARD1*, *ATM*, *PALB2*, *BRIP1*) and DNA mismatch repair and polyposis syndrome (*MUTYH*, *MLH1*, *PMS2*, *MSH6*, *APC*, *PMS2*). Detailed genotyping result, demographics, and histologic correlates of patients with pathogenic germline co-mutations are summarized in Table 2.

**Table 2:** Clinicopathologic features in 10 patients with *gBRCA* alterations with pathogenic or likely pathogenic co-mutations in other genes.

BRCA gene with mutation	Co-mutation genotype	Year of molecular diagnosis	Age of diagnosis	Race/Ethnicity	Histology
BRCA1	*MUTYH* (c.652G>T) heterozygous	2016	35	Hispanic or Latino	Cervical HSIL
BRCA1	*MUTYH* (c.1437_1439del) heterozygous	2006	49	White	Uterine carcinosarcoma
BRCA1	*PALB2* (c.2255_2267dup)	2020	72	White	Ovarian cancer/histology unknown
BRCA1	*MLH1* (c.1499delT)	2018	50	Asian	Endometrial carcinoma, endometrioid type
BRCA1	*SDHA* (c.1663+1G>T)	2018	46	White	Benign
BRCA1	*MUTYH* (c.934-2A>G) heterozygous	2019	45	Asian	Ovarian high grade serous
BRCA1	*CHEK2* (c.470T>C)	2018	46	White	Ovarian high grade serous
BRCA1	*APC* (c.3920T>A)	2018	54	White	Benign
BRCA1	*CDKN1C* (c.189_190insTTCCAGCTGG)	2017	69	Hispanic or Latino	Ovarian high grade serous
BRCA2	*PMS2* (c.1738A>T)	2018	47	Asian	Ovarian endometrioid

## Discussion

We summarize the results from our retrospective cohort study of unselected individuals with *BRCA1/2* germline mutations who were treated at our institution for gynecologic conditions. In our multiethnic population, Hispanic or Latino and Asian patients comprised 45% of our cohort, supporting that germline mutations are represented across all racial and ethnic groups. The major histologic subtype associated with *BRCA* mutations was tubo-ovarian high grade serous carcinoma, in keeping with the literature findings and clinical experience [1]. In our cohort, the g*BRCA* mutation prevalence in women with tubo-ovarian neoplasm (benign and malignant) without primary uterine malignancy was 6% (157/2844) [1]. Most of the testing was performed after 2013 (73% of our cohort), consistent with expansion and accessibility of molecular genetic testing due to multiple factors [9,10]. Overall, a significant number of patients in our *gBRCA* cohort was comprised of Hispanics or Latinos and Asians, contributing to the literature scarcity regarding genetic mutations in these patient populations. While it is known that *BRCA* mutations occur among all races and ethnicities, disparities in discussion of genetic testing by providers and election of genetic testing by patients have been noted in previous studies [11–13]. Our data supports discussion of genetic testing with all eligible patients, regardless of race or ethnicity.

Another interesting finding from our retrospective analysis is the increased use of multigene panel. From its usage, we identified 10 patients (5 Whites, 3 Asians, 2 Hispanic or Latino) with germline pathogenic co-mutations in various pathways (*MUYTH*, *PMS2*, *PALB2*, *MLH1*, *CHEK2*, *APC*, *CDKN1C*, and *SDHA*). This is a new finding that may have significant clinical implications that we will explore further in future research studies.

While our study confirmed that tubo-ovarian high grade serous carcinoma was the most associated malignant histology, there were other unexpected findings. Specifically, we found that the most common benign ovarian histology was serous cystadenoma and teratoma. Uniquely, one of the teratoma cases in our series exhibited somatic squamous cell carcinoma transformation, occurring in 43-year-old premenopausal women with *gBRCA2* mutation. This represents 20% (1 of 5) of the teratoma cases in our germline *gBRCA* patient cohort. By contrast, in routine settings, teratomas are generally benign, with malignant transformation being a rare event occurring in only 1–2% of cases, primarily in the postmenopausal setting [14,15]. While our finding occurred in a small sample size, future evaluation of relationship between *gBRCA* mutation and malignant transformation of teratoma, especially occurring in pre-menopausal setting, is warranted.

Another interesting histologic finding is presence of endometrioid histology, which is not typically associated with *BRCA* germline mutations [16]. In our *gBRCA* cohort, there were 8 patients who presented with endometrioid histology, occurring in either perimenopausal or postmenopausal age. Of these, 5 occurred as uterine cancer (3 pure endometrioid, 1 mixed endometrial and serous, 1 carcinosarcoma with endometrioid component) and 3 occurred as ovarian cancer (2 pure endometrioid, 1 mixed endometrioid and serous). Germline multigene panels were performed on 4 of the 8 patients who presented with endometrioid histology (the remaining 4 only received *BRCA1/2* gene analysis). Concurrent pathogenic germline mutations in *MLH1*, *PMS2*, *and MUTYH* were found in three patients (two in uterine cancer and one in ovarian cancer). It is unclear which germline mutation (mismatch repair gene or *BRCA*) is the dominant driver of endometrioid histology in this context. It is unclear which germline mutation (mismatch repair gene or BRCA) is the dominant driver of endometrioid histology in this context. However, MUTYH mutation is predicted to be less contributory due to its recessive nature. Furthermore, recent studies have reclassified the variant c.652G>% to be likely benign [17], as well as c.934-2A>G based on RNA and clinical data [18]. Further investigation is needed as multigene panel usage becomes more prevalent. Our finding suggests that endometrioid tumor histology does not exclude the possibility of underlying *gBRCA* mutation.

Although causality remains to be explored, the wide variety of histologic subtypes seen in individuals with germline *BRCA* mutations in our study could represent a larger spectrum of associated gynecologic conditions. Therefore, it may be prudent to consider germline genetic testing in individuals who have gynecologic cancers beyond high-grade serous cancer, especially in the setting of suspicious personal and/or family history. These findings also resonate with the recent debates regarding the optimal approach to germline testing: Should testing be restricted by personal or family history, by tumor histology and national guidelines, or universal panel-based approach [19]? It has been reported that testing restricted by personal or family history can lead to missing germline variant carriers [20,21]. Furthermore, testing guidelines are often family-history based and may disproportionately lead to underdiagnoses in some racial or ethnic populations [22]. While it’s tempting to adapt a universal panel-approach to avoid missing potential patients, the risks and health benefits of testing (e.g. whether the result will impact cancer treatment or overall outcomes) still need to be determined. Further studies may provide insight into how a universal panel-based approach could shift the epidemiologic and clinical data on germline *BRCA* patients in multiethnic population cohorts.

Approximately 57.4% of *BRCA1/2* mutations in our cohort were small insertions/deletions, most of which led to frameshift mutations. A pathogenic frameshift mutation usually introduces a premature stop codon, leading to nonfunctional truncated protein. We believe that reporting of *BRCA* genotype may have relevance for therapy prediction. For example, many ovarian cancer patients eventually develop poly(ADP)-ribose polymerase (PARP) inhibitors and platinum resistance via reversion mutations in *BRCA1/2*. Reversion mutations are secondarily acquired, often by small deletions, which restores the *BRCA1/2* allele reading frame, and therefore protein function [23]. This underscores the potential prognostic importance of reporting *BRCA* genotype and understanding the mutation type during oncologic management with PARP inhibitors. In our cohort, we found ~ 5% *gBRCA* patients with structural alteration. This subpopulation is theoretically “resistant” to developing reversion mutation in BRCA gene and may have more durable response with PARP inhibitors. Future epidemiologic studies should include mutation types so the association between *BRCA* genotype/mutation type and PARP inhibitor therapy response could be investigated.

Our study has several limitations. First, we do not have the test rate for our entire cohort, hence the estimate for *gBRCA* mutation prevalence may be an underestimated. This is due to lack of seamless integration of germline information with the rest of electronic medical records. The patients were identified by ICD-10 codes, which can either miss or include several patients. We attempted to rectify this shortcoming by having our extracted data manually inspected by three reviewers. Another limitation of our study is the low number of Black patients in our cohort. The exact cause is unknown, but likely multi-factorial. For example, cancer care and women’s health disparity in Black American is well-documented in the literature [8,11,24]. Further confounding variables include lower testing rate in this racial group [8]. Another possibility is that our hospital’s geographic location is inhabited by predominantly Asian and Hispanic or Latino population, leading to disproportional representation of racial groups. Additionally, our study uses race and ethnicity, which are socially, not genetically, defined traits. As such, although our data do provide support that any individual can have an inherited *BRCA* mutation, they cannot be used to comment on ancestry-specific mutation rates.

## Conclusion

To our knowledge, this is the first study to report the prevalence of germline pathogenic co-mutations in a cohort of women with *gBRCA* mutation with gynecologic condition. Future studies comparing the differences in the oncologic outcomes between patients with and without germline co-mutations are warranted. Our study also underscores the importance of using multigene panel in the evaluation of germline status, especially in the setting of additional personal and/or family history. This is becoming more feasible as the cost of genetic testing becomes more affordable, and the price differential between a small and large panel becomes smaller [25]. Further, knowing the presence of other germline mutations may lead to changes in treatment plan and could alter cancer screening and other medical interventions for the patient and relatives. As panels expand, incidental findings in genes not consistent with the reported history also become more common. As such, it is important to establish a nuanced and interdisciplinary approach to informed consent and genetic testing for patients.

## Supporting information

S1 Table *BRCA1/2* mutation status by race and ethnicity. Details of BRCA1/2 variants by type and race-ethnicity are presented.
(PDF)
