# Supplementary material for: BRCA germline mutations in multiethnic gynecologic patients: A 10-year retrospective analysis from a single cancer institute
Source: PLoS One. 2023 Jun 13;18(6):e0286998. doi: 10.1371/journal.pone.0286998 (PMC10263324; doi:10.1371/journal.pone.0286998)
Supplement: S1 Table — Details of BRCA1/2 variants by type and race-ethnicity are presented. (PDF) [file pone.0286998.s001.pdf]

Supplemental Table 1. BRCA1/2 Mutation Status by Race and Ethnicity.

Gene	*Mutation*	*Race/Ethnicity*
BRCA1	c.4065_4068del	Hispanic or Latino, non-Hispanic White
BRCA1	c.3770_3771del	Asian
BRCA1	c.1960A>T	Hispanic or Latino x2
BRCA1	c.3748G>T	Non-Hispanic White
BRCA1	c.3226dup	Asian
BRCA1	c.2504dup	Asian
BRCA1	c.5266dup	Non-Hispanic White x7
BRCA1	c.5155_5159delinsAAA	Asian
BRCA1	c.4243del	Non-Hispanic White
BRCA1	c.5095C>T	Non-Hispanic White
BRCA1	c.3598C>T	Hispanic or Latino x2
BRCA1	c.5137del	White
BRCA1	c.5123C>A	Hispanic or Latino x2
BRCA1	Whole gene deletion	Hispanic or Latino
BRCA1	c.3851_3852dup	Hispanic or Latino
BRCA1	c.68_69del	Non-Hispanic White x5, Hispanic or Latino x3, Asian, Unknown
BRCA1	c.5324T>G	Black
BRCA1	c.4524G>A	Black
BRCA1	C.212+1G>A	Hispanic or Latino x2
BRCA1	Deletion of exon 2	Non-Hispanic White
BRCA1	c.131G>A	Hispanic or Latino

BRCA1	c.784C>T	Asian
BRCA1	Deletion exons 8-11	Hispanic or Latino x3
BRCA1	c.4335_4338dup	Asian
BRCA1	c.1175_1214del	Non-Hispanic White
BRCA1	Deletion exons 1-7	Non-Hispanic White
BRCA1	c.798_799del	Hispanic or Latino x2
BRCA1	c.4327C>T	Hispanic or Latino x3
BRCA1	c.5354A>C	Non-Hispanic White
BRCA1	c.2433del	Unknown
BRCA1	c.1953_1956del	Non-Hispanic White
BRCA1	c.2389_2390del	Non-Hispanic White
BRCA1	c.3113_3114del	Non-Hispanic White
BRCA1	c.68_69dup	Hispanic or Latino
BRCA1	c.122A>G	Non-Hispanic White
BRCA1	c.4035del	Unknown
BRCA1	c.3747dup	Non-Hispanic White
BRCA1	c.5165C>A	Hispanic or Latino
BRCA1	c.213-11T>G	Non-Hispanic White
BRCA1	c.2035A>T	Non-Hispanic White
BRCA1	c.3756_3759del	Hispanic or Latino, Non-Hispanic White
BRCA1	c.3759_3760del	Hispanic or Latino
BRCA1	c.3964A>T	Non-Hispanic White
BRCA1	c.4888G>T	Asian

BRCA1	c.181T>G	Non-Hispanic White
BRCA1	c.1673_1674del	Non-Hispanic White
BRCA1	c.3436_3439del	Non-Hispanic White
BRCA1	c.211A>G	Hispanic or Latino x2
BRCA1	c.5353C>T	Non-Hispanic White
BRCA1	Duplication Exon 3	Hispanic or Latino
BRCA1	c.2014A>T	Asian
BRCA1	c.2806_2809del	Hispanic or Latino
BRCA1	c.3477_3480del	Non-Hispanic White
BRCA1	c.53T>C	Non-Hispanic White
BRCA1	c.3648dup	Non-Hispanic White
BRCA1	c.3481_3491del	Non-Hispanic White
BRCA1	c.2864C>A	Hispanic or Latino
BRCA1	c.134+1G>C	Asian
BRCA1	c.188T>A	Asian
BRCA1	c.349_350del	Hispanic or Latino
BRCA1	c.5080G>T	Non-Hispanic White
BRCA1	Unknown	Non-Hispanic White x12, Hispanic or Latino x3, Asian x2, Unknown x5
BRCA2	c.8167G>C	Non-Hispanic White x2
BRCA2	c.3264dup	Hispanic or Latino x6
BRCA2	c.2808_2811del	Non-Hispanic White x2, Unknown

BRCA2	Duplication exon 20	Non-Hispanic White
BRCA2	c.6944_6947del	Non-Hispanic White
BRCA2	c.5682C>G	Non-Hispanic White x2
BRCA2	c.4464_4465del	Hispanic or Latino
BRCA2	c.6428C>G	Non-Hispanic White
BRCA2	c.3847_3848del	Non-Hispanic White
BRCA2	c.5722_5723del	Hispanic or Latino
BRCA2	c.5217_5224del	Non-Hispanic White
BRCA2	c.7007G>A	Non-Hispanic White
BRCA2	c.4037_4038del	Hispanic or Latino
BRCA2	c.9117G>A	Hispanic or Latino
BRCA2	c.2037_2038del	Asian
BRCA2	c.7757G>A	Black
BRCA2	c.4936_4939del	Non-Hispanic White
BRCA2	c.2244C>G	Non-Hispanic White
BRCA2	c.4631del	Asian
BRCA2	c.700del	Non-Hispanic White
BRCA2	5471dup	Non-Hispanic White
BRCA2	c.5946del	Non-Hispanic White x3
BRCA2	c.8489G>A	Hispanic or Latino
BRCA2	c.6275_6276del	White
BRCA2	c.156_157insAlu	Hispanic or Latino
BRCA2	c.1755_1759del	Non-Hispanic White
BRCA2	c.3109C>T	Asian x2

BRCA2	c.6037A>T	Non-Hispanic White x2
BRCA2	c.1796_1800del	Asian
BRCA2	c.8487+1G>A	Asian
BRCA2	c.9317G>A	Asian
BRCA2	c.1754del	Non-Hispanic White
BRCA2	c.9025del	Black
BRCA2	c.5851_5854dup	Hispanic or Latino
BRCA2	c.5791C>T	Non-Hispanic White
BRCA2	c.3860dup	Hispanic or Latino
BRCA2	c.250C>T	Non-Hispanic White
BRCA2	c.5350_5351del	Non-Hispanic White
BRCA2	c.9382C>T	Hispanic or Latino
BRCA2	c.3170_3174del	Hispanic or Latino, Non-Hispanic White
BRCA2	c.4478_4481del	Non-Hispanic White
BRCA2	c.771_775del	Hispanic or Latino
BRCA2	c.3744_3747del	Asian
BRCA2	c.8322dup	Hispanic or Latino
BRCA2	c.7976+1G>A	Non-Hispanic White
BRCA2	c.68-2A>G	Non-Hispanic White
BRCA2	Unknown	Non-Hispanic White x7, Hispanic or Latino x4, Asian x2, Unknown
